# Automatic Work-Hours Recorder for Medical Staff (Staff Hours): Mobile App Development

**DOI:** 10.2196/16063

**Published:** 2020-02-25

**Authors:** Ting-Wei Chiang, Si-Yu Chen, Yuan-Chien Pan, Yu-Hsuan Lin

**Affiliations:** 1 Institute of Population Health Sciences National Health Research Institutes Miaoli County Taiwan; 2 Department of Psychology National Taiwan University Taipei Taiwan; 3 Department of Psychiatry National Taiwan University Hospital Taipei Taiwan; 4 Department of Psychiatry, College of Medicine National Taiwan University Taipei Taiwan; 5 Institute of Health Behaviors and Community Sciences, College of Public Health National Taiwan University Taipei Taiwan

**Keywords:** smartphone, mobile apps, medical staff, global positioning system, shift work schedule

## Abstract

**Background:**

There are numerous mobile apps for tracking work hours, but only a few of them record work hours automatically instead of relying on manual logging. No apps have been customized for medical staff, whose work schedules are highly complicated as they have both regular hours and on-call duties.

**Objective:**

The specific aims of this study were to (1) identify the *Staff Hours* app users’ GPS-defined work hours, (2) examine the overtime work hours from the app-recorded total work hours and the participants’ self-reported scheduled work hours, and (3) compare these app-recorded total work hours among different occupations.

**Methods:**

We developed an app, *Staff Hours*, to automatically calculate a user’s work hours via GPS background data. Users can enter their scheduled hours, including regular hours and on-call duties. The app automatically generates overtime reports by comparing the app-recorded total work hours with the user-defined scheduled hours. A total of 183 volunteers (60 females and 123 males; mean age 32.98 years, SD 6.74) were included in this study. Most of the participants (162/183, 88.5%) were medical staff, and their positions were resident physicians (n=89), visiting staff (n=38), medical students (n=10), registered nurses (n=25), and non–health care professionals (non-HCPs; n=21).

**Results:**

The total work hours (mean 55.69 hours, SD 21.34) of the 183 participants were significantly higher than their scheduled work hours (mean 50.67 hours, SD 21.44; *P*=.01). Medical staff had significantly longer total work hours (mean 57.01 hours, SD 21.20) than non-HCPs (mean 45.48 hours, SD 20.08; *P*=.02). Residents (mean 60.38 hours, SD 18.67) had significantly longer work hours than visiting staff (mean 51.42 hours, SD 20.33; *P*=.03) and non-HCPs (mean 45.48 hours, SD 20.08; *P*=.004).

**Conclusions:**

*Staff Hours* is the first automatic GPS location–based app designed for medical staff to track work hours and calculate overtime. For medical staff, this app could keep complete and accurate records of work hours in real time, reduce bias, and allow for better complying with labor regulations.

## Introduction

### Background

Long work hours and shift work increase the risk of both physiological and psychological distress [1-6], including cardiovascular disease [7] and depressive symptoms [8]. Medical staff, especially trainee physicians, work for a large number of hours per week and have a heavy workload. Systematic reviews have reported that such long working shifts and erratic schedules result in numerous adverse consequences in patient care [9]. In 2003, the Accreditation Council for Graduate Medical Education implemented the first work-hour restriction policy for all physicians in training in the United States, limiting resident physicians’ workweeks to 80 hours and shifts to 30 hours [10]. In Taiwan, similar policies by the Ministry of Health and Welfare limited workweeks to 88 hours in 2013 [11]. However, surveying medical interns’ compliance with the 2003 work-hour limits in the United States using a traditional assessment took 2 years; the resulting national survey was published in 2006 [10]. In addition, these self-reports do not reflect the fluctuations of work hours in real time, especially for medical staff with frequent on-call duties.

### Prior Work

Nowadays, smartphones offer us an objective and ecological source of measurement that continuously and passively collects data [12]. These reliable, quantitative data could facilitate real-time policy evaluation and target resources to those with the greatest need for them, even in remote and inaccessible regions. As of July 2019, the Google Play Store and iPhone Operating System (iOS) App Store had about 500 mobile apps dealing with the tracking of work hours [13]. However, most of those apps require manual entries or active clocking in and out. Fewer than 20 of those apps are capable of automatic timekeeping using various technologies such as GPS geofencing or Wi-Fi detections. Furthermore, medical personnel’s work hours could not be identified solely by GPS geofencing technology. Physicians often have several on-call duties every month. During their on-call duty, physicians have to be physically present at the hospital or be on-call at home. In addition, some medical staff practice in more than 1 institution, and these patterns of work hours could not be identified by a simple GPS geofencing algorithm.

### Goal of This Study

We herein report the design of the app *Staff Hours*, which automatically calculates users’ work hours through GPS data. Automatic recording of exact working hours is highly beneficial for people with erratic work schedules. This GPS-based work-hours recorder would not need users to precisely log their work hours by manual input or clocking in and out. The specific aims of this study were to (1) identify the app users’ GPS-defined work hours, (2) examine the overtime work hours from the app-recorded total work hours and the participants’ self-reported scheduled work hours, and (3) compare these app-recorded total work hours among different occupations.

## Methods

### Participants

We collected data on 183 office workers from July 30, 2018, to August 25, 2019, using the *Staff Hours* database. All participants were volunteers interested in their work hours, and workers who lived within a 1-km radius from their workplaces were excluded from this study. Of these participants, 123 were males and 60 were females, and their mean age was 32.98 (SD 6.74) years. Most of the participants (162/183, 88.5%) were medical staff, and their positions were resident physicians (n=89), visiting staff (n=38), medical students (n=10), registered nurses (n=25), and non–health care professionals (non-HCPs; n=21). All clinical investigations were conducted according to the principles expressed in the Declaration of Helsinki.

### Design of the Staff Hours App

*Staff Hours* is a mobile app designed to record work hours automatically using the technology of geofencing. As medical staff, along with most office workers, work at fixed locations, geolocation data are valid indicators of whether one is at work. On the basis of this principle, we developed algorithms to log the exact work hours from GPS data; these hours are called *GPS-defined work hours*. Furthermore, this app can perform overtime calculations by comparing the regular work schedule defined by the user and the corresponding GPS results.

*Staff Hours*, which was designed and developed by our team, is now available exclusively in Taiwan through the iOS App Store and the Google Play Store. The app runs in the background and records GPS information every 10 min with low power consumption. For iOS users, the app should be kept *in use* to function correctly, either in the foreground or background.

#### Algorithm for GPS-Defined Work Hours

**Figure 1:**
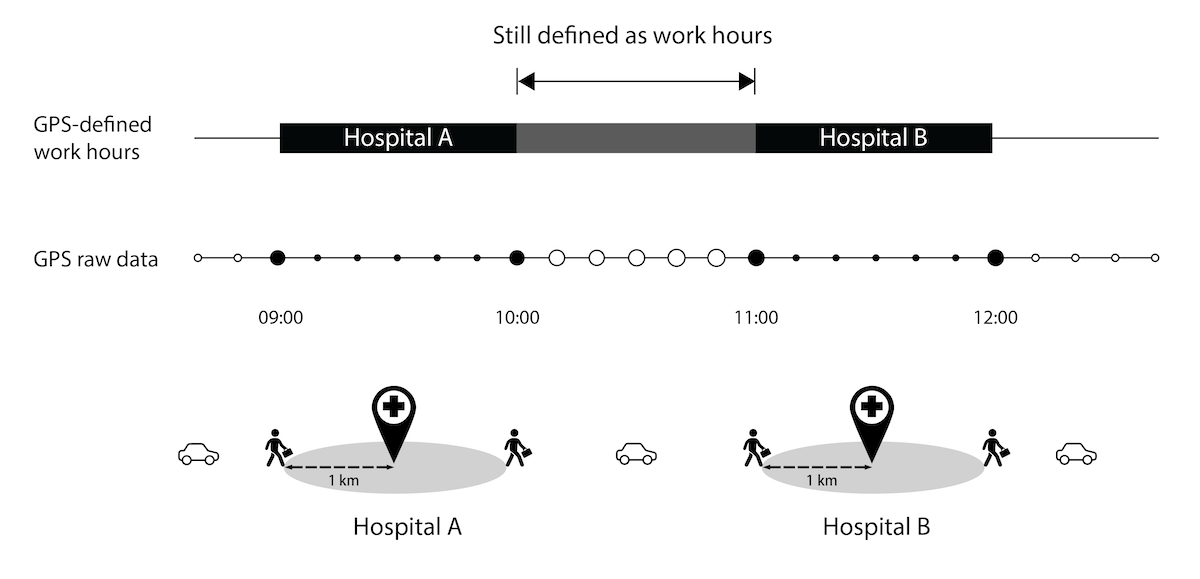
The recording process of GPS-defined work hours. For GPS raw data, the time interval between each dot is 10 min. If the coordinates captured are within the range, the dot is marked as solid; hollow if otherwise.

Upon installation, users would be asked to fill in the addresses of their workplaces; the app can track up to 5 locations simultaneously. Figure 1 illustrates how the app scans at 10-min intervals to detect whether the user’s GPS coordinates are within a 1-km radius centered on either of the workplaces. When a user has been at the workplace for 30 min, the app automatically starts recording the work hours, counting from the time that the user’s first location data within the range were received. Likewise, when a user leaves the neighborhood of the office for 30 min, the app considers the user to have gotten off work since the time that the first location data outside the range are collected.

#### Integration of Work Periods

**Figure 2:**
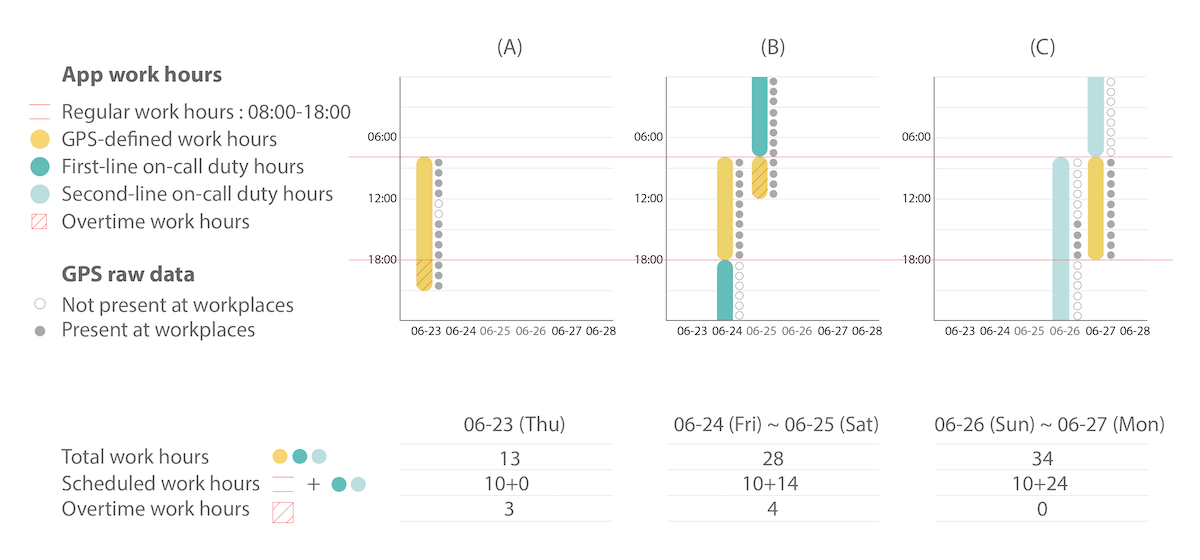
All types of work hours. (A) The regular work hours and overtime. (B) The on-call duty on a weekday and overtime. (C) The on-call duty on a weekend.

For any period in which a user is outside the GPS-detection range (considered not at work), if less than 8 hours, the app automatically integrates it with the former and the latter working periods, thus extending the GPS-defined work hours. In this context, when the user moves between the 5 or fewer recorded workplaces, or when the user leaves the current workplace but comes back later, the user is still considered *at work* for the whole time. The GPS-defined work hours would not be discontinued or interrupted in this case (Figures 1 and 2).

In the example in Figure 1, the user walks into and leaves the 1-km radius range of hospital A at 9 am and 10 am, respectively. First, 4 consecutive solid dots are collected at 9:30 am; so, the app records that the user has started working since 9 am. When 4 consecutive hollow dots are later collected at 10:30 am, the recording ends, resulting in a GPS-defined working period from 9 am to 10 am. Similar procedures are done as the user moves to work in hospital B; the generated GPS-defined working period in hospital B is from 11 am to 12 pm. Second, the app automatically integrates the detached GPS-defined working periods if the interval between them is less than 8 hours. As the gap between the working periods of hospitals A and B is only 1 hour (10:00-11:00), the app integrates them. Finally, the app logs the GPS-defined work hours as 3 hours (9:00-12:00).

#### Calculating Work Hours

**Figure 3:**
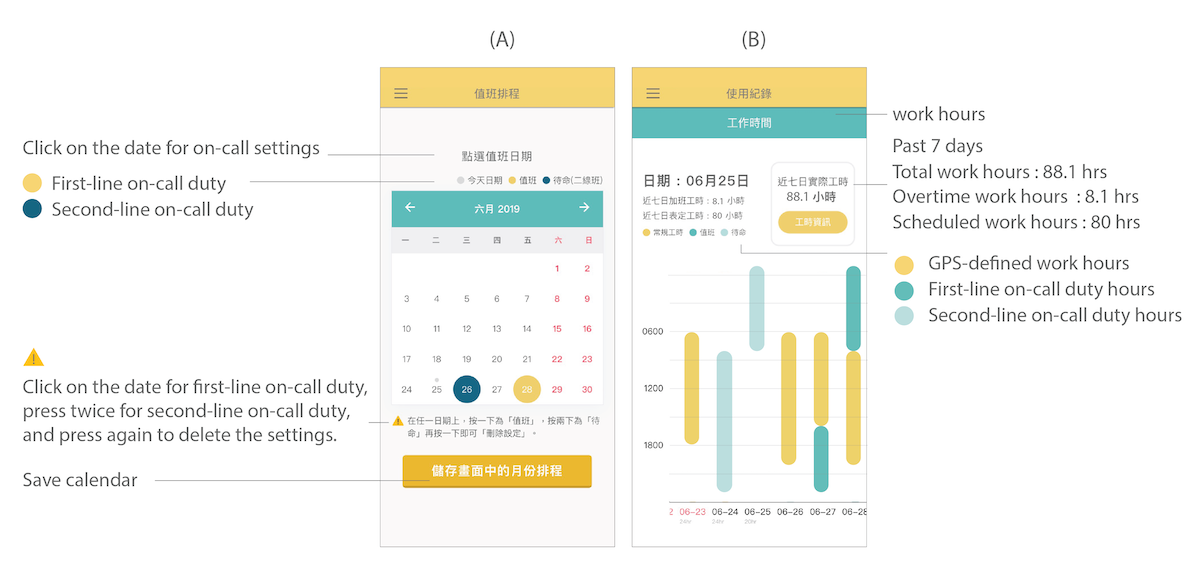
Screenshots of app user interface. (A) The on-call dates settings. (B) The work-hour statistics and charts.

Although the app counts GPS-defined work hours, users can also input their work schedules. The user-defined *scheduled work hours* include *regular work hours* and *on-call duty hours* (Figure 2). For regular work hours, users should set a fixed starting and finishing time of work for weekdays upon installation; for on-call hours, users should input the dates in a month on which they have on-call duties (Figure 3). The app offers 2 selections for on-call duty hours, in line with the standard policies of the hospitals in Taiwan. Medical staff with *first-line on-call duty* are obligated to stay in the hospital overnight to care for patients and respond to any situations. Others with *second-line on-call duty* are on standby, that is, they are not required to be on site but should be prepared to take phone calls anytime and return to the hospital if needed. For weekdays, we define *on-call duty hours* as the time from getting off regular work to that of starting regular work on the next day. On weekends, the staff should be on-call for 24 hours, beginning from the time of starting regular work.

The app automatically calculates the *total work hours* for users based on the following principles. During regular work time, the user’s total work hours are equal to the GPS-defined work hours with the integration algorithm, regardless of the regular work schedule. This is because one’s actual positioning data dominate the fixed work schedule. However, when it comes to on-call duties, the on-call hours have precedence over the GPS-defined hours because sometimes the staff is not obliged to be physically present within the range of the workplaces when working. In addition to the total work hours, this app also outputs the *overtime work hours* according to the following formula: *overtime work hours=total work hours−scheduled work hours.* The result can be a negative value, which would indicate that the exact working time was less than the scheduled working time (Figure 2).

The example in Figure 2 demonstrates how total and overtime work hours were calculated from the user’s work schedule and the recorded GPS data. The user set 8 am to 6 pm to be regular work hours (10 hours) from Monday to Friday. Thus, on-call duty hours on the picked dates should be from 6 pm to 8 am (+1) for weekdays (14 hours) and from 8 am to 8 am (+1) for weekends (24 hours). On June 23, the user worked for a total of 13 hours according to the integrated GPS-defined work hours despite a short break at noon. The period from 6 pm to 9 pm was regarded as overtime as it was beyond the scheduled regular hours. On June 24, the user had first-line on-call duty. Although absent from workplace from 6 pm to 0 am (+1), the whole period of 14 hours was still included in *total work hours* as the on-call duty hours took precedence over the actual hours detected at workplaces. As for Saturday, June 25, the user did not have regular work; so, it was considered overtime from 8 am to 12 pm. On Sunday, June 26, the user had second-line on-call duty lasting 24 hours and then continued with Monday’s regular work for another 10 hours. Hence, the total work hours were the sum of the 2 listed above, which equaled to 34 hours.

The users can check their total work hours, overtime work hours, and scheduled work hours for the past 7 days on the app. For higher accuracy and better flexibility, the app allows users to adjust their actual work hours within 7 days. In the example in Figure 3, the user had 8.1 hours of overtime in the past 7 days as it was the difference between the total work hours (88.1 hours) and scheduled work hours (80 hours). A visualization of a 28-day bar chart shows the staff’s total work hours, with yellow and green representing GPS-defined work hours and on-call hours, respectively. They can also share visualized data of their weekly or monthly average work hours on their Facebook page.

### Validation of App-Recorded Total Work Hours

We recruited 5 medical doctors and interviewed them regarding their work hours for the past 7 days to examine the accuracy of these (n=5×7=35) app-recorded total work hours by *Staff Hours*. This structural interview detailed the users’ on-call duties, regular work hours, and overtime work hours for each day. These self-reported total work hours excluded the trainee physicians’ educational activities. Therefore, we used these self-reported total work hours as the putative gold standard to validate the app-recorded total work hours. The sensitivity was 94.6% (242.7/256.6) and the specificity was 93.9% (547.6/583.4) for the app-recorded total work hours. There was a high correlation between the app-recorded total work hours and the self-reported counterparts (*r*=0.923; *P*<.001).

### Statistical Analysis

A paired *t* test was used to compare the differences between the total work hours and the scheduled work hours. One-way analysis of variance was used to compare the differences in total work hours among all 5 categories of workers (ie, medical students, visiting staff, resident physicians, registered nurses, and non-HCPs). Fisher least significant difference (LSD) was used for further post hoc tests. In addition, we used independent *t* tests to examine the differences in the total work hours between the medical staff (ie, medical students, visiting staff, resident physicians, and registered nurses) and non-HCPs. All statistical analyses were 2-tailed, and *P*<.05 was considered to be statistically significant. Data arrangement and statistical analysis were performed using R software (version 3.5.3).

## Results

### Overview

The app-recorded total work hours (mean 55.69 hours, SD 21.34) of all the 183 participants were significantly higher than the self-reported scheduled work hours (mean 50.67 hours, SD 21.44; *t*_182_=2.61; *P*=.01). All participants showed an average of 4.78 hours (SD 26.12) overtime work per week (ie, total work hours−scheduled work hours). Table 1 demonstrates that resident physicians had a significant positive overtime (mean 7.95 hours, SD 24.25), that is, the residents’ total work hours were significantly higher than the scheduled work hours (*t*_88_=3.14; *P*=.002).

**Table 1:** Total work hours and scheduled work hours among 5 occupations.

Occupation	Total work hours per week, mean (SD)	Scheduled work hours per week, mean (SD)
Resident physicians	60.38 (18.67)	52.31 (19.97)
Registered nurses	56.72 (28.80)	56.89 (30.88)
Visiting staff	51.42 (20.33)	44.46 (17.43)
Medical students	49.03 (20.06)	45.95 (13.79)
Non-HCPs	45.48 (20.08)	49.83 (22.17)

Table 1 also shows the comparisons of total work hours and scheduled work hours among the 5 categories. There were significant differences among the 5 categories on total work hours (*F*_4,183_=3.047; *P*=.02). Post hoc comparisons using LSD tests further revealed that the resident physicians had significantly longer total work hours (mean 60.38 hours, SD 18.67) than the visiting staff (mean 51.42 hours, SD 20.33; *P*=.03) and non-HCPs (mean 45.48 hours, SD 20.08; *P*=.004). In addition, medical staff (ie, resident physicians, registered nurses, visiting staff, and medical students) had significantly longer total work hours (mean 57.01 hours, SD 21.20) than non-HCPs (mean 45.48 hours, SD 20.08; *P*=.02).

## Discussion

### Principal Findings

Our findings showed that medical staff had longer work hours than non-HCPs, and resident physicians worked for longer hours than visiting staff in hospitals. These findings are consistent with previous reports that showed that resident physicians typically work the greatest number of hours per week among all positions in hospitals [14]. Similarly, it is feasible to compare work hours among different hospitals, departments, or divisions using aggregate data collected from the Staff Hours’ database. In this way, we can find out the *hot spots* where office workers have the longest total work hours or overtime. From September 2019, the Labor Standards Act in Taiwan will include regulations on the working hours for resident physicians. With the implementation of government policy, *Staff Hours* can serve as a real-time monitor for compliance with these work-hour limits. The app might also be useful in labor negotiations, balancing the unequal information between employers and employees. Although employers hold official records on work hours, the automatically recorded data in mobile apps provide evidence for employees when they claim overtime pay. In this study, each occupational group comprised greater than or equal to 10 people, which strengthens user privacy. Further analysis of the comparisons among different clusters should take individual privacy into account and make sure that there are not less than 10 people in each group.

The algorithms behind *Staff Hours* include not only the location-based work hours recording but also several critical designs for a better user experience. First, detached working periods are linked back together in the GPS-defined work hours because of the unique duty-based characteristics of health care work. Take a physician at an academic hospital as an example. She may have to leave the hospital for lectures on the university campus or walk a couple of blocks in the neighborhood to grab lunch in the middle of the scheduled working time. If the physician comes back to the hospital in less than 8 hours, the app still records the period that she is absent from the GPS range of the hospital as GPS-defined work hours. Second, the update interval of GPS location data in this app is set to be 10 min to improve battery performances; this is much longer than the geofencing responsiveness interval of approximately 2 min in the background location limits introduced in Android 8.0 (Application Program Interface level 26) to reduce undesired battery drain [15]. Third, users can adjust the work-hour logs manually only within 7 days. This restriction was established to avoid recall bias, increasing the accuracy of records [16]. Another associated advantage is that it may assure data integrity and prevent future problems of data fraud, which would be crucial in labor inspections.

In addition, this app performs functions that specifically target medical staff, such as automatic calculations of overtime and on-call duty hours. Our findings demonstrated that all the participants on average worked 4.78 more hours per week than scheduled. The lengthy overtime may be from a selection bias in our participants, most of whom (89/183, 48.6%) were resident physicians whose total work hours were always higher than they should be. However, we observed that the scheduled ones were shorter than expected because few residents (58/89, 65%) logged their on-call dates in the system, which might result in an underestimation of the scheduled work hours. Moreover, this app could not differentiate the activities of working and learning in the institution for residents and medical students, which might result in an overestimation of total work hours. Together, they may lead to an overestimation of overtime and thus, we observed significant differences between the total and scheduled work hours in this study. Unsure of the validity of the scheduled and overtime work-hours data, we analyzed only the total work hours of the various occupations.

### Limitations

Several methodological limitations should be noted when interpreting our findings. First, users who live within a 1-km geofencing radius from their workplaces are limited to using the algorithm of GPS-defined work hours. Furthermore, inaccuracies in GPS location tracking may occur when users work at smaller companies or clinics as the app transforms workplace addresses into latitudes and longitudes. Second, if only a few medical staff log their on-call duties in the app, the produced average overtime hours could be higher than the actual overtime hours. Third, the app could not identify breaks within the geofencing range, which might result in an overestimation of total work hours. Finally, there may be a selection bias in the participants as medical staff or other users with longer work hours may have a higher tendency to install *Staff Hours*.

### Comparison With Prior Work

*Staff Hours* is the first app designed specifically for medical staff, with built-in functions for tracking on-call duties in hospitals. To the best of our knowledge, no prior study has enabled medical staff to use their personal smartphones, either Apple or Android models, to monitor their work hours automatically. The high accuracy of GPS-defined work-hours data without manual input and the integration between multiple work locations show the app’s ease of use. Moreover, *Staff Hours* is the first app to perform comparisons on work hours among different categories of medical staff and also between medical staff and other occupations. For staff having excessive work hours, this app could serve as a *smoke alarm*, providing early signals of overwork or occupational burnout. We designed this app focusing on medical staff’s work-hour patterns as they may have the most complicated work-hour patterns, including frequent on-call duties and multiple workplaces. The power-saving GPS data collection with optimal sampling rate and work periods integration in our algorithm overcame these challenges.

### Conclusions

*Staff Hours* is the first automatic GPS location–based app designed for medical staff to track work hours and calculate overtime. For medical staff, this app could keep complete and accurate records of work hours in real time, reduce bias, and allow for better complying with labor regulations.

## Abbreviations

iOS: iPhone Operating System
LSD: least significant difference
non-HCP: non–health care professional
